# Glycosylated haemoglobin and prognosis in 10,536 people with cancer and pre-existing diabetes: a meta-analysis with dose-response analysis

**DOI:** 10.1186/s12885-022-10144-y

**Published:** 2022-10-06

**Authors:** Suping Ling, Michael Sweeting, Francesco Zaccardi, David Adlam, Umesh T. Kadam

**Affiliations:** 1 Department of Health Sciences, University of Leicester, University Road, Leicester, LE1 7RH UK; 2 Present address: Department of Non-communicable Disease Epidemiology, Faculty of Epidemiology and Population Health, London School of Hygiene & Tropical Medicine, Keppel St, London, WC1E 7HT UK; 3 Leicester Real World Evidence Unit, Leicester Diabetes Centre, University of Leicester, Leicester, UK; 4 Leicester Diabetes Centre, University of Leicester, Leicester, UK; 5 Department of Cardiovascular Sciences and NIHR Leicester Biomedical Research Centre, University of Leicester, Leicester, UK

**Keywords:** Diabetes, cancer prognosis, cancer survival, Mortality, HbA_1c_, Glycaemic control, Systematic review, Dose-response meta-analysis

## Abstract

**Aims:**

To assess whether glycaemic control is associated with prognosis in people with cancer and pre-existing diabetes.

**Methods:**

In this pre-registered systematic review (PROSPERO: CRD42020223956), PubMed and Web of Science were searched on 25th Nov 2021 for studies investigating associations between glycosylated haemoglobin (HbA_1c_) and prognosis in people with diabetes and cancer. Summary relative risks (RRs) and 95% Confidence Intervals (CIs) for associations between poorly controlled HbA_1c_ or per 1-unit HbA_1c_ increment and cancer outcomes were estimated using a random-effects meta-analysis. We also investigated the impact of potential small-study effects using the trim-and-fill method and potential sources of heterogeneity using subgroup analyses.

**Results:**

Fifteen eligible observational studies, reporting data on 10,536 patients with cancer and pre-existing diabetes, were included. Random-effects meta-analyses indicated that HbA_1c_ ≥ 7% (53 mmol/mol) was associated with increased risks of: all-cause mortality (14 studies; RR: 1.14 [95% CI: 1.03–1.27]; *p-value*: 0.012), cancer-specific mortality (5; 1.68 [1.13–2.49]; *p-value*: 0.011) and cancer recurrence (8; 1.68 [1.18–2.38; *p-value*: 0.004]), with moderate to high heterogeneity. Dose-response meta-analyses indicated that 1-unit increment of HbA_1c_ (%) was associated with increased risks of all-cause mortality (13 studies; 1.04 [1.01–1.08]; *p-value*: 0.016) and cancer-specific mortality (4; 1.11 [1.04–1.20]; *p-value*: 0.003). All RRs were attenuated in trim-and-fill analyses.

**Conclusions:**

Our findings suggested that glycaemic control might be a modifiable risk factor for mortality and cancer recurrence in people with cancer and pre-existing diabetes. High-quality studies with a larger sample size are warranted to confirm these findings due to heterogeneity and potential small-study effects. In the interim, it makes clinical sense to recommend continued optimal glycaemic control.

**Supplementary Information:**

The online version contains supplementary material available at 10.1186/s12885-022-10144-y.

## Highlights

• Diabetes is a common comorbidity in newly-diagnosed cancer patients.

• The impact of glycaemic control in people with both cancer and diabetes is unclear.

• In this meta-analysis, cancer prognosis is worse in those with poor HbA_1c_ control.

• More studies with larger sample sizes are warranted to confirm these findings.

• Clinicians should continue to ensure HbA_1c_ control in cancer patients with diabetes.

**Supplementary Information:**

The online version contains supplementary material available at 10.1186/s12885-022-10144-y.

## Introduction

Cancer is an important cause of death worldwide. The Global Burden of Cancer Study reported an estimated 19 million new cancer cases and 10 million cancer deaths worldwide in 2020 [1]. Comorbidity, a potential determinant of cancer treatment, is becoming increasingly common in cancer patients, driven in part by an ageing population [2]. In particular, diabetes has become one of the most common comorbidities in cancer patients [3]. One Danish study reported that 7% of breast, 10% of prostate, 13% of colon and bladder, 25% of pancreatic, and 30% of liver cancer patients had diabetes at cancer diagnosis [4]. One of the reasons could be the shared risk factors (e.g., obesity, poor diet, physical inactivity) and common biological mechanisms between cancer and diabetes [5]; diabetes itself has been recognised as a potentially aetiological factor for many cancers [6, 7].

Several meta-analyses have shown that, compared to those without, cancer patients with pre-existing diabetes had a worse prognosis [8–10]. Among the proposed biological pathways, hyperglycaemia can stimulate tumour growth, thereby leading to disease progression [11]. A meta-analysis also reported that hyperglycaemia in solid tumours is associated with worse overall survival, regardless of the presence of diabetes diagnosis [12]. With the rising prevalence of diabetes globally, [13] the number of cancer patients with comorbid diabetes is expected to increase. While robust evidence from both randomised controlled trials and observational studies indicates a progressive association between glucose levels and risk of long-term cardiovascular diseases in people with diabetes, [14, 15] less is known about the relationship between glycaemic control, as measured by glycosylated haemoglobin (HbA_1c_), and prognosis in patients with cancer and pre-existing diabetes.

In this meta-analysis with dose-response analysis, we summarised the current evidence on the association between HbA_1c_ and cancer prognosis in people with both cancer and diabetes.

## Materials and methods

### Data sources and search strategy

We followed the Preferred Reporting Items for Systematic reviews and Meta-Analyses (PRISMA) guideline for this study [16] and registered the study protocol within PROSPERO (No. CRD42020223956). On 25th Nov 2021, we systematically searched MEDLINE (via PubMed) and Web of Science for observational cohort studies or post-hoc analyses of clinical trials in cancer patients with diabetes that reported the association between HbA_1c_ and cancer prognosis, including: mortality, cancer recurrence, cancer progression, and hospitalisations. The search was limited to records in English. Keywords related to diabetes, HbA_1c_ or glycaemia, cancer, and prognosis were used in the search. Bibliographies of relevant reviews were additionally sought to identify eligible studies. Details of the search strategy and the PRISMA checklist are shown in the Supplementary Material.

### Study selection and data extraction

All titles and abstracts were independently screened by two reviewers (SL and UTK); articles with any disagreement at this stage were included for full-text assessment. Studies were eligible if they reported the relative risk (RR) estimate (hazard ratio, risk ratio, or odds ratio) with their standard errors (SEs), 95% confidence intervals (CIs), or *p*-values for the association between HbA_1c_ and cancer prognosis; SEs were calculated from 95% CIs or *p*-values if not reported [17]. Studies were excluded if: (1) not all subjects had cancer and diabetes; (2) the exposure was not HbA_1c_ (e.g., fasting glucose). If two or more articles included the same participants, the analysis with largest person-years was included. If a study was stratified by cancer, estimates for different sites were treated as different cohorts.

A standardised form was used to extract data on the study characteristics, participants, cancer sites, definitions and ascertainment of exposures and outcomes, mean/median of HbA_1c_, number of participants, events and person-years, duration of follow-up, methods of analysis, and most-adjusted estimates for each outcome. If no estimate was reported but Kaplan-Meier curves was available, we firstly extracted data from the curves using Engauge Digitizer and then used the “*ipdfc*” command in Stata to reconstruct individual-level time-to-event data from curves and applied Cox proportional hazard model to estimate the hazard ratio [18].

### Risk of bias assessment

We used the Newcastle-Ottawa Scale (NOS) to assess the quality of included studies. This scale quantifies the risk of bias in observational studies based on three domains: selection of population, comparability, and ascertainment of outcomes. In the comparability domain, age and cancer characteristics were defined as the two most important factors that studies should adjust for. In the outcome domain, less than 20% loss to follow-up was deemed adequate; the sufficient length of follow-up was determined by the severity of cancer (e.g., 1 year is considered sufficient for pancreatic, 3 years for bladder, and 5 years for prostate cancer). Based on these criteria, each study was assigned a score ranging from 0 (lowest quality) to 9 (highest).

### Statistical analysis

Our primary analyses sought to combine the RRs for the associations between poorly and well controlled HbA_1c_, with 7% (53 mmol/mol) as the glycaemic target according to current diabetes management guidelines [19]. Based on data availability, where possible we converted comparisons into poorly (≥ 7%) vs. well controlled HbA_1c_ (< 7%); the flowchart for data conversion is reported in Supplementary Fig. 1. If a study reported associations between continuous HbA_1c_ and outcomes, estimates were converted into comparisons above vs. below the cut-off (≥7% vs < 7%) as described in Chene et al. [20] If, conversely, a study reported the comparison across other cut-offs, the effect for per 1-unit increment was firstly estimated and then converted into comparisons ≥7% vs. < 7% [21, 22]. The secondary analysis aimed to quantify the dose-response relationship between HbA_1c_ and outcomes. Some studies were not included in this analysis as means/medians of HbA_1c_ were not reported and therefore it was not possible to convert estimates for categories to per 1-unit increment.

Due to inconsistent terminology and definitions of end-points, we classified cancer prognosis outcomes into: all-cause mortality, cancer-specific mortality, and cancer recurrence (including local, regional and distant recurrence, and/or metastasis) following guidelines for time-to-event end-point definitions in cancer studies [23].

Summary RRs and 95% CIs for poorly controlled HbA_1c_ and per 1-unit increment of HbA_1c_ (%) were combined using a random-effects model [24]. Heterogeneity across studies was quantified by the *I*^*2*^ statistics: we deemed an *I*^*2*^ value of lower than 50% as low, 50 to 75% as moderate, and larger than 75% as high [25]. Small-study effects (e.g., publication bias) were assessed by funnel plots and the Egger’s test [26]. We further investigated the impact of potential small-study effects using the trim-and-fill method and potential sources of heterogeneity using subgroup analyses. We used Stata/IC version 16.0 (Stata Corp, College Station, TX) for all analyses and considered a two tailed *p-value* < 0.05 as statistically significant.

## Results

### Characteristics of the included study

**Fig. 1:**
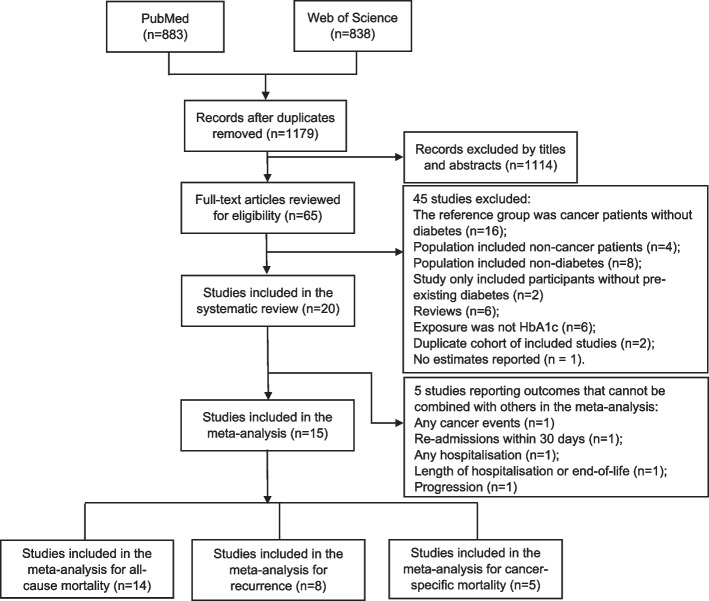
Flowchart of study selection

We identified 1179 papers in the systematic search; after screening of titles and abstracts, 65 records met the eligible criteria for full-text assessment: of these, 20 studies reported data on the associations between HbA_1c_ and prognosis but five studies reported the outcome which could not be combined with other studies. Therefore, we included 15 studies with 10,536 participants with cancer and pre-existing diabetes in the meta-analysis. The flowchart of study selection is shown in Fig. 1; reasons and references for the excluded studies are presented in Supplementary Table 1.

The characteristics of the included studies are presented in Table 1. Overall, more than two-thirds of the studies were from Asian countries and the sample size was generally small (*N* < 500), except in two studies from UK and US (*N* > 1000). The most commonly investigated cancer site was bladder (*n* = 6), followed by pancreas (*n* = 3); most studies reported estimates for cut-offs of HbA_1c_, such as 7%, while some reported per 1-unit increment of HbA_1c_ (details in Supplementary Table 2). The median follow-up ranged from 9 months to 6.8 years and only one-third of studies adjusted for at least one confounding factor. The overall quality of the included studies was moderate, with NOS scores ranging from 4 to 8 (median, 7; Supplementary Table 3).

**Table 1:** Characteristics of included studies

Study	Country	N^a^	Cancer	Is HbA_**1c**_ the main exposure	analysis of HbA_**1c**_	Median follow-up	outcomes	ascertainment of outcomes
Ahn et al. (2016) [27]	Korea	127	Bladder	Yes	HbA_1c_ ≥ 7% vs. < 7%	50 months	Cancer recurrence-free survival; progression-free survival	RFS was defined as the time from initial TUR to first tumor recurrence (regardless of grade or stage),PFS was define as the time from initial TUR to tumor progression, any increase in grade (G1/2 to G3) or stage (Ta to T1 or T2, T1 to T2) after repeat TUR for recurrence.
Boursi et al. (2016) [28]	UK	7916	Bladder, breast, colorectal, pancreatic, prostate	Yes	Continuous HbA_1c_	4.1 years (bladder); 4.4 (breast); 3.3 (colorectal); 1.2 (pancreatic); 4.2 (prostate)	Overall survival	All-cause death
Cheon et al. (2014) [29]	Korea	65	Pancreatic	Yes	HbA_1c_ ≥ 7% vs. < 7%	9 months	Overall survival	All-cause death
Huang et al. (2020) [30]	Taiwan, China	33	Bladder	Yes	HbA_1c_ ≥ 7% vs. < 7%	45 months	Cancer recurrence-free survival	RFS was defined as the period from the date of the initial TUR of bladder tumour to the date of the operation in which the first cancer recurrence was found.
Hwang et al. (2011) [31]	Korea	92	Bladder	No	HbA_1c_ ≥ 7% vs. < 7%	30 months	Cancer recurrence; cancer progression	RFS was defined as the time from initial TUR to first tumour recurrence (regardless of grade or stage), whereas PFS was defined as the time from initial TUR to tumour progression (any increase in grade [G1/2 to G3] or stage [Ta to T1 or T2, T1 to T2]) after repeat TUR for recurrence.
Kaneda et al. (2012) [32]	Japan	26	HCC	Yes	HbA_1c_ ≥ 6.5% vs. < 6.5%	23 months	Cancer recurrence	NR
Kang et al. (2016) [33]	Korea	135	Bladder	Yes	HbA_1c_ ≥ 7% vs. < 7%	33.8 months	Cancer recurrence-free survival; Bladder cancer specific survival; overall survival	RFS, CSS, and OS, were defined from the date of surgery to the date of recurrence, death from upper tract urothelial carcinoma, and death from any cause
Komatsu et al. (2020) [34]	Japan	259	Lung	No	Continuous HbA_1c_; HbA_1c_ ≥ 7% vs. < 7%	39 months	Overall survival	All-cause death
Lee et al. (2016) [35]	Korea	77	Pancreatic	Yes	HbA_1c_ ≥ 9% vs. < 9%	20 months	Overall survival; disease-free survival	Disease-free survival: cancer recurrence or death
Lee et al. (2017) [36]	Korea	61	Colon	Yes	HbA_1c_ ≥ 8% vs. < 8%	NR	Overall survival	All-cause death
Li et al. (2017) [37]	China	89	Cervical	Yes	continuous HbA_1c_; HbA_1c_ ≥ 7% vs. < 7%	39 months	Cancer recurrence-free survival; cancer specific mortality; overall survival	RFS, CSS and OS were calculated from the date of neoadjuvant chemotherapy until the date of events (recurrence or death from cervical cancer or death from any cause)
Nik-Ahd et al. (2019) [38]	USA	1409	Prostate	Yes	Continuous HbA_1c_;	6.8 years	Cancer metastases; cancer recurrence; overall survival; prostate-specific cancer mortality	Metastases were defined as the first metastasis determined from any type of imaging test.
Okamura et al. (2017) [39]	Japan	64	Oesophageal	Yes	HbA_1c_ ≥ 7% vs. < 7%	NR	Overall survival; disease-specific mortality	OS and disease-specific survivals were calculated from either surgery to death or last follow-up
Siddiqui et al. (2008) [40]	USA	155	Colorectal	Yes	HbA_1c_ ≥ 7.5% vs. < 7.5%	NR	overall survival; cancer-specific mortality	All-cause death; cause-specific death
Tai et al. (2015) [41]	Taiwan, China	55	Bladder	Yes	HbA_1c_ ≥ 7% vs. < 7%	51 months	Cancer recurrence-free survival	Cancer recurrence referred to tumour relapse in operative filed, regional lymph nodes and/or distant metastasis.

*HbA*_*1c*_ Glycosylated haemoglobin, *TUR* Transurethral resection, *RFS* Recurrence-free survival, *PFS* Progression-free survival, *CSS* Cancer-specific survival, *OS* Overall survival

^a^N: total number of participants; NR: not reported or cannot be estimated based on reported data

### Poorly versus well controlled HbA_1c_

**Fig. 2:**
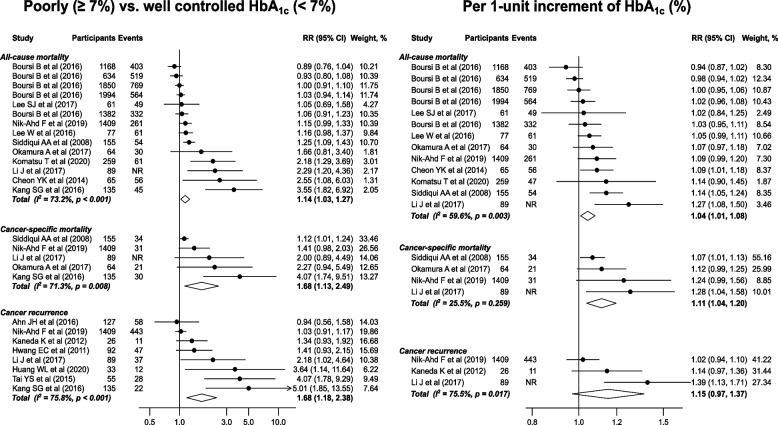
Pooled associations between HbA_1c_ and outcomes. Study-specific and overall estimates for poorly controlled HbA_1c_ [≥ 7% (53 mmol/mol)] compared to well controlled HbA_1c_ [< 7% (53 mmol/mol)] [left] and per 1-unit increment of HbA_1c_ (%) [right]. NR: not reported in the original study

**Fig. 3:**
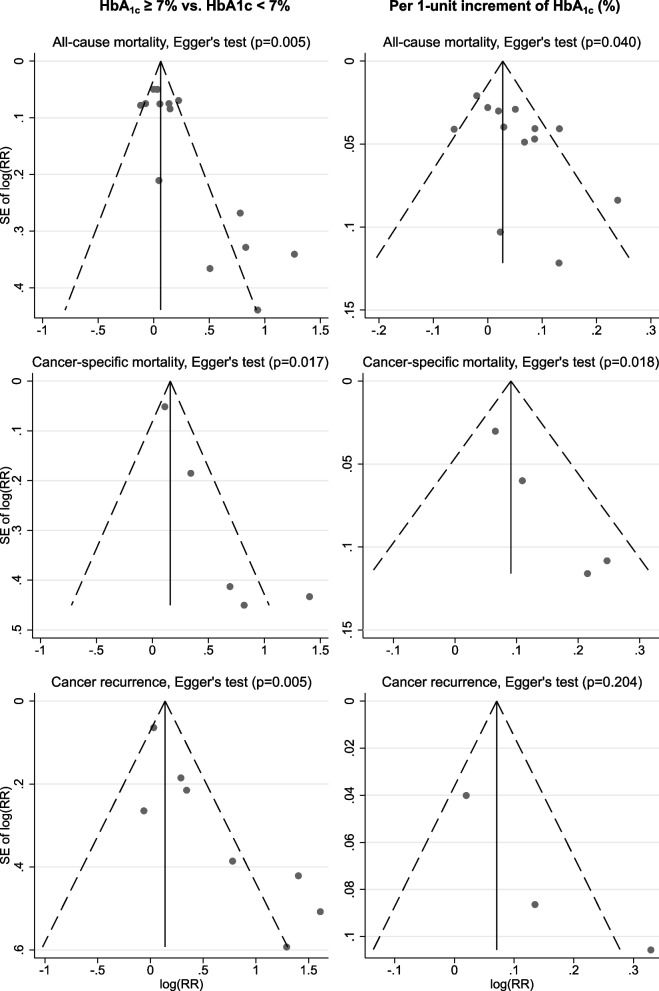
Small-study effects. Funnel plots reported for poorly controlled HbA_1c_ [≥ 7% (53 mmol/mol)] compared to well controlled HbA_1c_ [< 7% (53 mmol/mol)] [left] and per 1-unit increment of HbA_1c_ (%) [right]

For the comparison of HbA_1c_ ≥ 7% vs <  7%, pooled meta-analytical estimates were obtained for all-cause mortality, cancer-specific mortality, and cancer recurrence. Random-effects meta-analyses suggested that, compared to HbA_1c_ <  7%, patients with HbA_1c_ ≥ 7% had an increased risk of all-cause mortality (14 studies; 9342 subjects and 3204 deaths; RR: 1.14; 95% CI: 1.03–1.27; *p* = 0.012), cancer-specific mortality (5 studies;1852 subjects and 116 cancer-specific deaths; 1.68; 1.13–2.49; *p* = 0.011) and cancer recurrence (8 studies; 1966 subjects, of which 658 with a cancer recurrence; 1.68; 1.18–2.38; *p* = 0.004), with moderate to high heterogeneity across studies for all three outcomes (*I*^*2*^ 73.2% and *p* < 0.001; 71.3% and *p* = 0.008; and 75.8% and *p* < 0.001, respectively; Fig. 2). Results of Egger’s test and funnel plots are shown in Fig. 3, indicating small-study effects for all three outcomes (Egger’s test *p* = 0.005, *p* = 0.017, and *p* = 0.005, respectively). Pooled RRs for all-cause mortality, cancer-specific mortality, and cancer recurrence in trim-and-fill analyses were attenuated to 1.06 (95% CI: 0.94–1.20; *p* = 0.355), 1.20 (0.83–1.74; *p* = 0.340), and 1.24 (0.87–1.78 *p* = 0.241) after imputing potential unpublished studies (Supplementary Table 4). Funnel plots for trim-and-fill analyses are presented in Supplementary Fig. 2.

### Secondary analysis: dose-response meta-analysis

Five studies reporting RRs of HbA_1c_ across a cut-off could not be converted into effects per 1-unit change (Supplementary Table 2). As shown in Figs. 2, 1-unit increment of HbA_1c_ (%) was associated with increased risks of all-cause mortality (13 studies; 9207 subjects and 3145 deaths; RR: 1.04; 95% CI: 1.01–1.08; *p* = 0.016) and cancer-specific mortality (4 studies; 1717 subjects and 86 cancer-specific deaths; 1.11; 1.04–1.20; *p* = 0.003) but not cancer recurrence (3 studies; 1524 subjects of which 443 with a cancer recurrence; 1.15; 0.97–1.37; *p* = 0.110). We observed moderate heterogeneity for all-cause mortality (*I*^*2*^ 59.6%; *p* = 0.003; Fig. 2) and potential small-study effects for both all-cause mortality (Egger’s test, *p* = 0.040) and cancer-specific mortality (*p* = 0.018; Fig. 3). Taken potential unpublished studies into account, the trim-and-fill analyses showed pooled RRs of 1.02 (95% CI: 0.98–1.06; *p* = 0.374) and 1.08 (1.00–1.17; *p* = 0.053) for all-cause mortality and cancer-specific mortality, respectively (Supplementary Table 4); Supplementary Fig. 2 shows the corresponding funnel plots.

### Subgroup analyses

We also conducted subgroup analysis by limiting the inclusion to studies of high quality (NOS score ≥ 6): results are shown in Supplementary Fig. 3. RRs were slightly attenuated for all-cause mortality but strengthened for cancer-specific mortality and cancer recurrence. For the comparison of poorly vs. well controlled HbA1c, the RR was 1.03 (95% CI: 1.00–1.07; *p* = 0.041) for all-cause mortality, 2.06 (1.11–3.82; *p* = 0.021) for cancer-specific mortality, and 1.71 (1.10–12.65; *p* = 0.018) for cancer recurrence. Heterogeneity across studies was slightly reduced for cancer-specific mortality but not for all-cause mortality or cancer recurrence.

Subgroup analyses by geographical areas were possible for all-cause mortality and cancer-specific mortality. We observed differences in the association of poorly vs. well controlled HbA_1c_ with all-cause mortality and cancer-specific mortality between Asian and Western studies (Supplementary Fig. 4a): the RR for all-cause mortality was 1.04 (95% CI: 0.96–1.12; *p* = 0.348 in 7 studies, 8592 subjects, and 2902 deaths) in Western while it was 1.14 (1.03–1.27; *p =* 0.001 in 7 studies, 750 subjects, and 302 deaths) in Asian studies (*p* < 0.01 for difference by subgroups). The RR for cancer-specific mortality was 1.17 (0.98–1.41; *p* = 0.089 in 2 studies, 1564 subjects, and 65 deaths) in Western and 2.63 (1.61–4.28; *p* < 0.001 in 3 studies, 288 subjects, and 51 deaths) in Asian studies (*p* < 0.01 for difference by subgroups). Estimates for per 1-unit increment of HbA_1c_ by geographical areas are shown in Supplementary Fig. 4b.

Analyses by cancer sites were possible for bladder, colorectal, pancreatic, and prostate cancer for all-cause mortality; and bladder cancer for recurrence (Supplementary Fig. 5). Moderate to high heterogeneity was observed in most subgroup analyses. A significant association between poorly vs. well controlled HbA_1c_ and bladder cancer recurrence was found in five studies reporting this association (442 subjects of which 167 with a cancer recurrence; RR: 2.23; 1.18–4.21; *p* = 0.013; Supplementary Fig. 5).

## Discussion

Our results, obtained from 15 studies with data on 10,536 patients with cancer and diabetes, showed that a poorly controlled diabetes and a progressively higher HbA_1c_ were associated with a worse cancer prognosis. We observed moderate to high heterogeneities across the included studies and small-study effects for most outcomes which may have biased the estimates. Subgroup analyses suggested that differences in the quality of studies, cancer sites, and geographical areas might have contributed to such heterogeneities. Notably, geographical differences was likely attributable to the smaller study sample sizes in Asia than Western countries, though other unexamined factors may have also contributed to such differences, such as earlier onset of diabetes (i.e., at younger ages) and/or at lower body mass index in Asia [42]. Further original investigations with a lager sample size are needed to confirm current findings.

Previous meta-analyses have shown a poor survival associated with diabetes in patients with cancer, [8] including prostate, [9] pancreatic, [10] breast, [43] cervical, [44] colorectal, [45] lung, [46] and brain [47] cancer. Although the exact mechanisms underpinning worse outcomes in cancer patients with comorbid diabetes are unknown, hyperglycaemia and hyperinsulinemia have been proposed as possible biological pathways due to their roles in stimulating tumour growth [11]. Another meta-analysis suggested a positive association between hyperglycaemia and mortality in patients with cancer, regardless of the presence of diabetes diagnosis [12]. While most studies, original investigations, or systematic reviews included people without diabetes as the comparison group, to our knowledge there was no meta-analysis on the association between glycaemic control (or HbA_1c_ levels) and survival in people with both cancer and diabetes. In particular, we found that HbA_1c_ was associated with cancer recurrence in patients with bladder cancer and pre-existing diabetes.

Previous meta-analyses of randomised controlled trials indicated that improved glycaemic control or additional weight change achieved by current glucose-lowering medications was not associated with cancer incidence, [48, 49] suggesting that these biological pathways alone cannot fully explain the anti-tumour effect of glycaemic control and other collateral effects related to diabetes and cancer management may also have a part to play. Indeed, the presence of poorly controlled diabetes may affect the timing of cancer diagnosis in both directions, which may determine the stage at cancer diagnosis. Cancer stage is one of the most important determinants of prognosis, with long-term survival being much greater in early stages [50]. On the one hand, patients with poorly controlled diabetes might have a more frequent healthcare contact, which would possibly lead to an earlier cancer detection [51]. On the other hand, poorly controlled diabetes may represent a group of patients requiring a high burden of care, leading to a “competing demand” of diabetes care and less awareness of cancer symptoms [52]. A population-based study using electronic health records in Canada suggested that, among newly-diagnosed breast cancer patients, compared to those without diabetes, individuals with diabetes presented with a higher stage and were more likely to have metastases [53]. However, whether glycaemic control is related to stage at cancer diagnosis in people with diabetes and cancer was not investigated in the current meta-analysis due to lack of information, and further studies with individual-level data are needed.

The selection of cancer treatment is also important for prognosis while it may be delivered differently based on the glycaemic control and further contributed to disparities in prognosis. Diabetes is one of the risk factors for infection in cancer patients, [54] and a meta-analysis suggested that cancer patients with diabetes were also at greater risk of postoperative mortality [55]. Hence, surgery may be postponed if HbA_1c_ is poorly controlled. Moreover, poorly controlled HbA_1c_ may lead health care professionals to reduce the doses/regimens of some treatments, as the use of steroids (a common treatment for many cancers) increases glucose levels [56]. In addition, some common chemotherapeutic agents and newer targeted therapies for cancer may potentially cause cardiotoxic complications [57]. While diabetes, particularly uncontrolled, is a major risk factor of cardiovascular diseases, [14, 15] it is possible that hyperglycaemia would make these people with cancer and pre-existing diabetes more susceptible to such complications. However, in the current meta-analysis information related to diabetes medications (some of which may have antineoplastic effects independent of risk factor control [58]) or cancer treatment was not available in most of the included studies and therefore warrants further investigations.

It should be noted that the magnitude of the increased risks of cancer associated with diabetes varied by cancer types: for example, although diabetes was associated with both pancreatic and bladder cancer, the relative risks were over 2.0 for pancreatic and 1.2 for bladder cancer [7]. In light of the heterogeneous survival in people with different cancer types (e.g., 5-year survival rates for bladder and pancreatic were 52.6 and 6.5% in England during 2013–2017 [59]), the effect of glycaemic control on cancer survival may differ among diabetic people with different cancers similar to the variable effect of diabetes on cancer incidence. In fact, albeit with small numbers of studies, our subgroup analyses would suggest potential different relative risks of all-cause mortality in people with bladder and pancreatic cancer but no inference could be obtained due to the limited data; future research should focus on specific cancers to detail such differences.

Our study has important clinical implications. The impact of cancer diagnosis and treatment on diabetes management has drawn less attention, possibly because both clinicians and cancer patients may prioritise cancer over glycaemic control after a cancer diagnosis [60]. Based on current evidence, clinicians should continue to ensure glycaemic control in people with cancer and pre-existing diabetes, and it should be integral to clinical cancer care. This is also reflected in guidelines on glycaemic control in people with cancer recently issued by The Joint British Society for Inpatient Care and UK Chemotherapy Board, which emphasises the important of glucose monitoring in all patients with cancer, regardless of their diabetic status [61, 62]. While these guidelines are provided to reduce the acute hyperglycaemia-related complications during cancer treatment periods (in short-term), [61] our study fills the gap by suggesting that ameliorating pre-existing hyperglycaemia could improve survival also in the long-term, though future studies with large sample sizes are warranted to identify the optimal glycaemic goal and medications in people with cancer.

Our study has also some strengths and limitations. To our knowledge, this study is the first meta-analysis investigating the prognostic role of HbA_1c_ in people with both cancer and diabetes; we also examined associations across a range of end-points, including all-cause mortality, cancer recurrence, and cancer-specific mortality, which are relevant in overall prognosis as well as in cancer epidemiology. To minimise the impact of publication bias, we extracted additional data from Kaplan-Meier curves if no estimates were reported. Yet, we still observed potential small-study effects for most outcomes, and RRs were attenuated to statistical non-significance in trim-and-fill analyses after imputing potentially unpublished studies. We have only included English articles which may have introduced language bias. The quality of included studies was moderate, particularly due to lack of adjustment for other important clinical factors (e.g., cancer characteristics, sex, body weight) which may have confounded the causal association between glucose control and the investigated outcomes. Age and cancer stage, for example, are the two most relevant confounders which have been adjusted for only in five studies; obesity itself is as an important risk factor for both diabetes and cancer, [5] and body weight may also affect the dose of chemotherapy. In addition, other diabetes related-factors, such as disease duration and treatment, may have also contributed to confounding. Furthermore, we included studies with heterogeneous prognosis and thus some of the statistical heterogeneity we observed was expected. Where possible, we performed subgroup analyses to detail cancer-specific associations. Nevertheless, due to limited data, we were not able to characterize the potentially diverse prognostic roles of HbA_1c_ in different cancer populations. We have also conducted subgroup analyses by geographical areas and the quality of study to investigate sources of heterogeneity: our findings indicated that both factors may have contributed to the observed heterogeneities. In particular, the opposite directions in the of associations accounting for small-study effects (attenuated) vs the inclusion of only studies of higher quality (strengthened) for some outcomes would suggest the relevant impact of publication bias and study quality on the interpretation of our findings. Heterogeneity could also be related, however, to other factors, such as demographics of included participants, cancer characteristics, diabetes and cancer treatment, which were not investigated because of lack of relevant information in the included studies. In line with the current diabetes management guidelines, we converted all estimates into comparisons of above vs below a clinically relevant threshold (7%; 53 mmol/mol) but it should be noted that these conversions were based on two assumptions: a normal HbA_1c_ distribution and a linear relationship with outcomes. Yet, we could not explore a potential non-linearity in the relationship between HbA_1c_ and outcomes; therefore, further research is warranted to identify the optimal glycaemic target for cancer patients with diabetes.

In conclusion, our meta-analyses suggests that poor glycaemic control may be associated with worse outcomes in patients with cancer and diabetes. However, current findings are limited by evidence of potential bias in the published literature and more high-quality studies with a larger sample size are needed to confirm these conclusions; in the interim, it makes clinical sense to recommend continued optimal glycaemic control based on current evidence and guidelines. Further investigations are also warranted to identify the optimal goal for glycaemic control and characterise the effect of HbA_1c_ in different cancer populations.

## Supplementary Information

**Additional file 1 **Search algorithms on 25th Nov 2021. **Supplementary Table 1.** Reasons of exclusion of studies following full-text review. **Supplementary Table 2.** Data conversions and references of included studies. **Supplementary Table 3.** Newcastle-Ottawa score for included studies. **Supplementary Table 4.** Trim-and-fill analyses results. **Supplementary Fig. 1.** Flowchart for data conversion. **Supplementary Fig. 2.** Funnel plots following trim-and-fill. **Supplementary Fig. 3.** Meta-analysis within studies of high quality (NOS score ≥ 6). **Supplementary Fig. 4a.** Subgroup analyses by geographical region for HbA1c ≥ 7% vs. HbA1c <  7%. **Supplementary Fig. 4b.** Subgroup analyses by geographical region per 1-unit increment of HbA1c. **Supplementary Fig. 5.** Subgroup analyses by cancer sites. PRISMA checklist.

## Data Availability

The datasets used and/or analysed during the current study available from the corresponding author on reasonable request. Data extracted from included studies will be available online if accepted; analytic codes used in the meta-analysis are available from https://github.com/supingling/HbA1c_cancer_meta-analysis.
