# Supplementary material for: Glycosylated haemoglobin and prognosis in 10,536 people with cancer and pre-existing diabetes: a meta-analysis with dose-response analysis
Source: BMC Cancer. 2022 Oct 6;22:1048. doi: 10.1186/s12885-022-10144-y (PMC9535893; doi:10.1186/s12885-022-10144-y)
Supplement: Supplementary file 1 — Additional file 1 Search algorithms on 25th Nov 2021. Supplementary Table 1. Reasons of exclusion of studies following full-text review. Supplementary Table 2. Data conversions and references of included studies. Supplementary Table 3. Newcastle-Ottawa score for included studies. Supplementary Table 4. Trim-and-fill analyses results. Supplementary Fig. 1. Flowchart for data conversion. Supplementary Fig. 2. Funnel plots following trim-and-fill. Supplementary Fig. 3. Meta-analysis within studies of high quality (NOS score ≥ 6). Supplementary Fig. 4a. Subgroup analyses by geographical region for HbA1c ≥ 7% vs. HbA1c < 7%. Supplementary Fig. 4b. Subgroup analyses by geographical region per 1-unit increment of HbA1c. Supplementary Fig. 5. Subgroup analyses by cancer sites. PRISMA checklist. [file 12885_2022_10144_MOESM1_ESM.pdf]

SUPPLEMENTARY MATERIAL

Glycosylated Haemoglobin and prognosis in 10,536 people with cancer and pre-existing diabetes: A meta-analysis with dose-response analysis

Suping Ling, Michael Sweeting, Francesco Zaccardi, David Adlam, Umesh T. Kadam

Contents

Search algorithms on 25th Nov 2021 2
Supplementary Table 1. Reasons of exclusion of studies following full-text review 3
Supplementary Table 2. Data conversions and references of included studies 4
Supplementary Table 3. Newcastle-Ottawa score for included studies 6
Supplementary Table 4. Trim-and-fill analyses results 7
Supplementary Figure 1. Flowchart for data conversion 8
Supplementary Figure 2. Funnel plots following trim-and-fill 9
Supplementary Figure 3. Meta-analysis within studies of high quality (NOS score ⩾ 6) 10
Supplementary Figure 4a. Subgroup analyses by geographical region for HbA_{1c} ⩾ 7% vs. HbA_{1c} < 7% 11
Supplementary Figure 4b. Subgroup analyses by geographical region per 1-unit increment of HbA_{1c} 12
Supplementary Figure 5. Subgroup analyses by cancer sites 13
PRISMA checklist 14
References 17

Search algorithms on 25th Nov 2021

PubMed (883 records)

(diabet*[title/abstract]) AND (glycated haemoglobin[title/abstract] OR glycosylated[Title/Abstract] OR A1c[Title/Abstract] OR HbA1c[Title/Abstract] OR glycohemoglobin[Title/Abstract] OR glycohaemoglobin[Title/Abstract] OR glycemi*[Title/Abstract] OR glycaemi*[Title/Abstract] OR hyperglycaemia[Title/Abstract] OR hyperglycemia[Title/Abstract]) AND (cancer[title/abstract] OR carcinoma[title/abstract] OR neoplasia[title/abstract] OR tumor[title/abstract] OR tumour[title/abstract] OR neoplasm[title/abstract] OR maligna*[title/abstract]) AND (prognosis[title/abstract] OR progression[title/abstract] OR hospitalis*[title/abstract] OR hospitaliz*[title/abstract] OR *admission*[title/abstract] OR surviv*[title/abstract] OR recurrence[title/abstract] OR mortality[title/abstract])

English and Humans only

Web of Science (838 records)

AB=(diabet*) AND AB=(glycated haemoglobin OR glycosylated OR A1c OR HbA1c OR glycohemoglobin OR glycohaemoglobin OR glycemi* OR glycaemi* OR hyperglycaemia OR hyperglycemia) AND AB=(cancer OR carcinoma OR neoplasia OR tumor OR tumour OR neoplasm OR maligna*) AND AB=(prognosis OR progression OR hospitalis* OR hospitaliz* OR admission* OR surviv* OR recurrence OR mortality)

Articles or proceeding papers only; English only.

Supplementary Table 1. Reasons of exclusion of studies following full-text review

Study	Reason for exclusion
Alpertunga, I., et al. (2021) [1]	Population included non-diabetes
Arrieta, O., et al. (2016) [2]	Exposure was not HbA_{1c}
Barua, R., et al. (2018) [3]	Reviews
Cantiello, F., et al. (2015) [4]	Reviews
Chang, Y. L., et al. (2018) [5]	The reference group was cancer patients without diabetes
Chen, S., et al. (2017) [6]	Reviews
Chen, X. H., et al. (2020) [7]	Exposure was not HbA_{1c}
Chia, C. L. K., et al. (2016) [8]	The reference group was cancer patients without diabetes
Choe, S., et al. (2020) [9]	Exposure was not HbA_{1c}
Connor, A. E., et al. (2019) [10]	Exposure was not HbA_{1c}
Ederaine, S. A., et al. (2021) [11]	The reference group was cancer patients without diabetes
Erickson et al (2011) [12]	The outcome was any cancer events, cannot be combined with others
Fan, K. Y., et al. (2014) [13]	Study only included participants without pre-existing diabetes
Ferroni, P., et al. (2016) [14]	Population included non-cancer patients
Hope, C., et al. (2016) [15]	Reviews
Hershey et al (2017) [16]	The outcome was hospitalisation, cannot be combined with others
Iavazzo et al (2016) [17]	The outcome was re-admission, cannot be combined with others
Joshu, C. E., et al. (2012) [18]	Population included non-cancer patients
Karlin, N. J., et al. (2018) [19]	The reference group was cancer patients without diabetes
Karlin, N. J., et al. (2018) [20]	The reference group was cancer patients without diabetes
Karlin, N. J., et al. (2018) [21]	The reference group was cancer patients without diabetes
Karlin, N. J., et al. (2017) [22]	The reference group was cancer patients without diabetes
Karlin, N. J., et al. (2020) [23]	The reference group was cancer patients without diabetes
Karlin, N. J., et al. (2019) [24]	The reference group was cancer patients without diabetes
Karlin, N. J., et al. (2012) [25]	The reference group was cancer patients without diabetes
Karlin, N. J., et al. (2019) [26]	The reference group was cancer patients without diabetes
Kaseda, K., et al. (2020) [27]	The reference group was cancer patients without diabetes
Kim, H. S., et al. (2010) [28]	Duplicate cohort of included studies
Kochi, R., et al. (2020) [29]	Population included non-diabetes
Kondo et al (2013) [30]	The outcome was length of hospitalisation, cannot be combined with others
Kurishima, K., et al. (2017) [31]	The reference group was cancer patients without diabetes
Lee, H., et al. (2015) [32]	Population included non-diabetes
Lee et al (2015) [33]	The outcome was unknown, cannot be combined with others
Li, J., et al. (2020) [34]	Population included non-cancer patients
Liang, S. H., et al. (2020) [35]	Duplicate cohort of included studies
Liu, H., et al. (2016) [36]	Reviews
Motoishi, M., et al. (2018) [37]	Population included non-diabetes
Murtola, T. J., et al. (2019) [38]	The reference group was cancer patients without diabetes
Onitilo, A. A., et al. (2013) [39]	Population included non-cancer patients
Ogawa, H., et al. (2021) [40]	Population included non-cancer patients
Pusceddu, S., et al. (2018) [41]	Exposure was not HbA_{1c}
Sandini, M., et al. (2020) [42]	Population included non-diabetes
Shi, H. J., et al. (2017) [43]	Population included non-diabetes
Shimada, S., et al (2021) [44]	Population included non-diabetes
Simon, J. M., et al. (2018) [45]	Study only included participants without pre-existing diabetes
Suceveanu, A. I., et al. (2020) [46]	No estimates reported
van Herpt, T. T. W., et al. (2011) [47]	The reference group was cancer patients without diabetes
Wrenn, S. M., et al. (2021) [48]	The reference group was cancer patients without diabetes
Zeng, X. H., et al. (2020) [49]	Exposure was not HbA_{1c}
Zhao, X. B. and G. S. Ren (2016) [50]	Reviews

Studies are sorted alphabetically.

Supplementary Table 2. Data conversions and references of included studies

Study	Original analysis	Adjustment	Data conversion	Parameters required	Parameters reported	Action
Ahn et al (2016) [51]	Univariable Cox	NA	Cut-off to continuous	Mean/Median of HbA1c	No	Not included in per 1-unit analysis
Boursi et al (2016) [52]	Multivariable Cox	Commonly adjusted for: obesity, smoking history, alcohol consumption, coronary artery disease (CAD), hypertension, hyperlipidaemia, Additionally adjusted for Colorectal: chronic NSAIDs/aspirin use and hormone replacement therapy. Breast: hormone replacement therapy. Bladder: chronic NSAIDs/aspirin use and oral anti-diabetes medications other than metformin.	Continuous to cut-off 7%	Mean/Median of HbA1c	Median/IQR of HbA1c of whole group	Assuming HbA1c normally distributed, calculated mean for two groups with cut-off of 7%; estimated HR for HbA1c ≥7% vs. <7%
Cheon et al (2014) [53]	Multivariable Cox	CA 19-9, chemotherapy, and antidiabetic treatment.	Cut-off to continuous	Mean/Median of HbA1c	Median/range of HbA1c of each group	Estimated HR for per 1-unit using the median of two groups
Huang et al (2020) [54]	Univariable Cox	NA	Cut-off to continuous	Mean/Median of HbA1c	No	Not included in per 1-unit analysis
Hwang et al (2011) [55]	Logistic regression*	NA	Cut-off to continuous	Mean/Median of HbA1c	No	Not included in per 1-unit analysis
Kaneda et al (2012) [56]	Univariable Cox*	NA	Cut-off 6.5% to continuous; continuous to cut-off 7%	Mean or SD of HbA1c	Median/10th/90th percentiles of HbA1c of whole group	Assuming HbA1c normally distributed, calculated mean/SD from median/percentiles; calculated the mean for two groups (cut-off 6.5%); estimated HR for per 1-unit using the mean of two groups; calculated the mean for two groups (cut-off 7%); estimated HR for HbA1c ≥7% vs. <7%
Kang et al (2016) [57]	Univariable Cox*	NA	Cut-off to continuous	Mean/Median of HbA1c	No	Not included in per 1-unit analysis
Komatsu et al (2020) [58]	Multivariable Cox*	Age, sex, smoking, BMI and stage	NA			HR is calculated directly from raw data (reported individual-level data)
Lee et al (2016) [59]	Univariable Cox*	NA	Cut-off 9% to continuous then continuous to cut-off 7%	Mean/Median of HbA1c	Mean/SD	Estimated HR per 1-unit using the mean of two groups (cut-off 9%); assuming HbA_{1c} normally distributed, calculated the mean for two groups (cut-off 7%); estimated HR for HbA_{1c} ≥7% vs. <7%

Study	Original analysis	Adjustment	Data conversion	Parameters required	Parameters reported	Action
Lee et al (2017) [60]	Multivariable Cox	Age, sex, WBC, CRP, total cholesterol, HDL, LDL, triglycerides	Cut-off 8% to continuous; continuous to cut-off 7%	Mean/Median of HbA1c	Mean/SD	Estimated HR per 1-unit using the mean of two groups (cut-off 8%); assuming HbA_{1c} normally distributed, calculated the mean for two groups (cut-off 7%); estimated HR for HbA_{1c} ≥7% vs. <7%
Li et al (2017) [61]	Multivariable Cox	Deep stromal invasion, margins, nodes, parametrium	NA			The study reported both cut-off 7% and per 1-unit; these estimates were included in the meta-analyses
Nik-Ahd et al (2019) [62]	Multivariable Cox	Biopsy grade group and prostate-specific antigen	Continuous to cut-off 7%	Mean/Median of HbA1c	Median/percentiles of HbA1c of whole group	Assuming HbA1c normally distributed, calculated mean/SD from median/IQR; calculated mean for two groups (cut-off 7%); estimated the HR for HbA1c >7% vs. <7%
Okamura et al (2017) [63]	Univariable Cox*	NA	Cut-off to continuous	Mean/Median of HbA1c	Median/range of HbA1c of each group	Estimated the HR per 1-unit using the median of two groups
Siddiqui et al (2008) [64]	Logistic regression	NA	Cut-off 7.5% to continuous; continuous to cut-off 7%	Mean/Median of HbA1c	Mean/SD of HbA1c of each group	Estimated the HR per 1-unit using the mean of two groups (cut-off 7.5%); calculated mean for two groups (cut-off 7%); estimated HR for HbA1c >7% vs. <7%
Tai et al (2015) [65]	Univariable Cox*	NA	Cut-off to continuous	Mean/Median of HbA1c	No	Not included in per 1-unit analysis

*Calculated from reported data. NA: Not applicable; NR: Not reported.

Supplementary Table 3. Newcastle-Ottawa score for included studies

Study	Selection				Compare		Outcome			Total
	a	b	c	d	e	f	g	h	i	
Ahn et al (2016)	1	1	1	1	0	0	1	1	1	7
Boursi et al (2016)	1	1	1	1	1	0	1	1	1	8
Cheon et al (2014)	1	1	1	1	0	1	1	0	1	7
Huang et al (2020)	1	1	0	1	0	0	0	1	1	5
Hwang et al (2011)	1	1	1	1	0	0	1	0	1	6
Kaneda et al (2012)	0	1	0	1	0	0	1	1	1	5
Kang et al (2016)	1	1	1	1	0	0	1	0	1	6
Komatsu et al (2020)	1	1	1	1	0	0	1	1	1	7
Lee et al (2016)	1	1	1	1	0	0	1	1	1	7
Lee et al (2017)	1	1	1	1	1	0	0	0	0	5
Li et al (2017)	1	1	1	1	0	1	1	1	1	8
Nik-Ahd et al (2019)	1	1	1	1	0	1	1	1	1	8
Okamura et al (2017)	1	1	1	1	0	0	0	0	0	4
Siddiqui et al (2008)	1	1	1	1	0	0	0	0	0	4
Tai et al (2015)	1	1	1	1	0	0	1	1	1	7

a Representativeness of the exposed
b Selection of the non-exposed
c Ascertainment of exposure
d Outcome was not present at start of study
e Controlled for age
f Controlled for stage
g Assessment of outcome
h Follow-up long enough
i Lost to follow-up

Supplementary Table 4. Trim-and-fill analyses results

Analysis	Outcome	Trim-and-fill	Pooled RR (95% CI)	Number of studies	
				Observed	Imputed
HbA_{1c} ≥ 7% vs. HbA_{1c} < 7%	All-cause mortality	No	1.14 (1.03, 1.27)	14	0
		Yes	1.06 (0.94, 1.20)	14	4
	Cancer-specific mortality	No	1.68 (1.13, 2.49)	5	0
		Yes	1.20 (0.83, 1.74)	5	3
	Recurrence	No	1.68 (1.19, 2.38)	8	0
		Yes	1.24 (0.87, 1.78)	8	3
Per 1-unit increment of HbA_{1c} (%)	All-cause mortality	No	1.04 (1.01, 1.08)	13	0
		Yes	1.02 (0.98, 1.06)	13	4
	Cancer-specific mortality	No	1.11 (1.04, 1.20)	4	0
		Yes	1.08 (1.00, 1.17)	4	2

RR: Relative risk

Supplementary Figure 1. Flowchart for data conversion

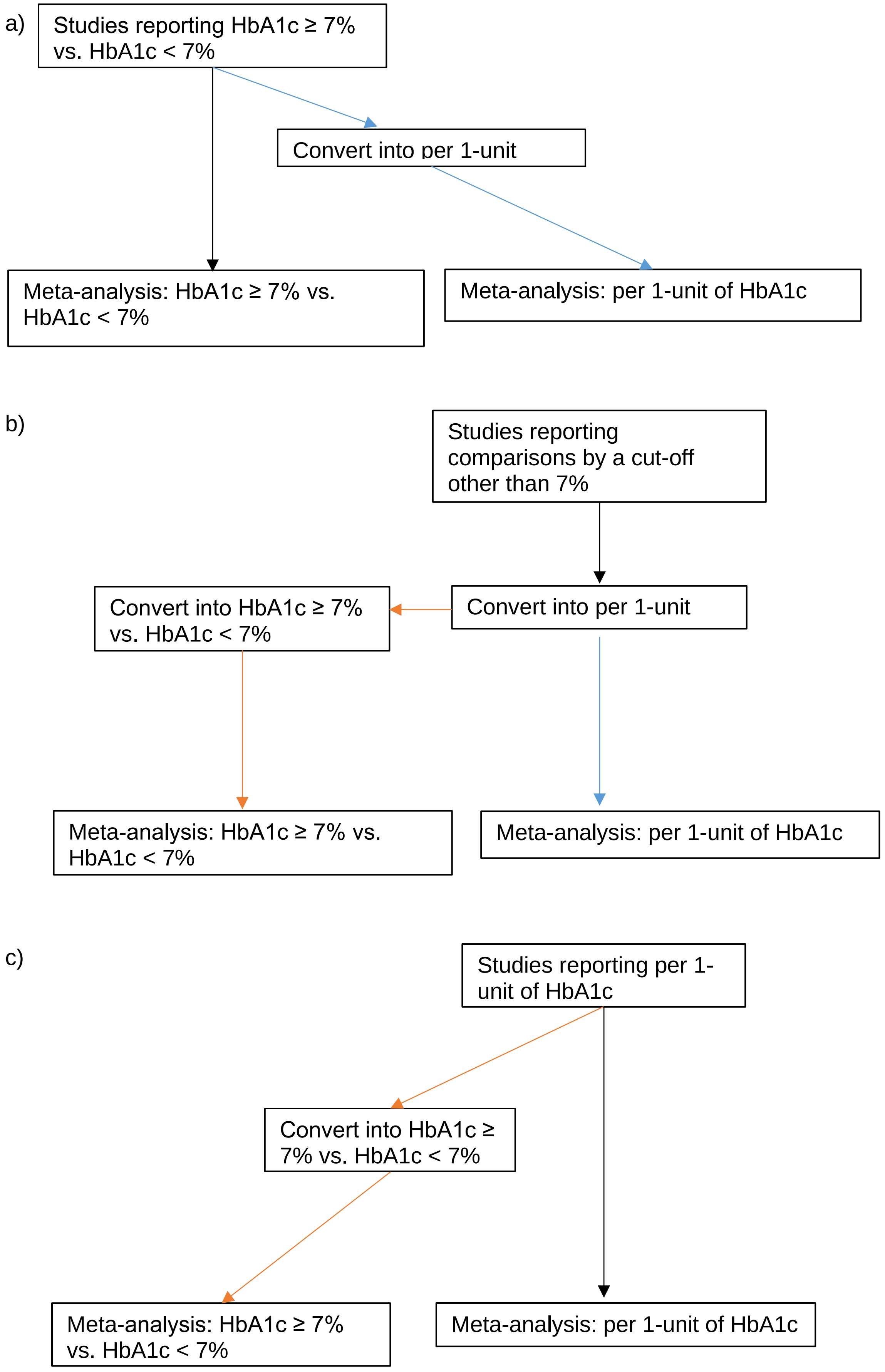

Supplementary Figure 2. Funnel plots following trim-and-fill

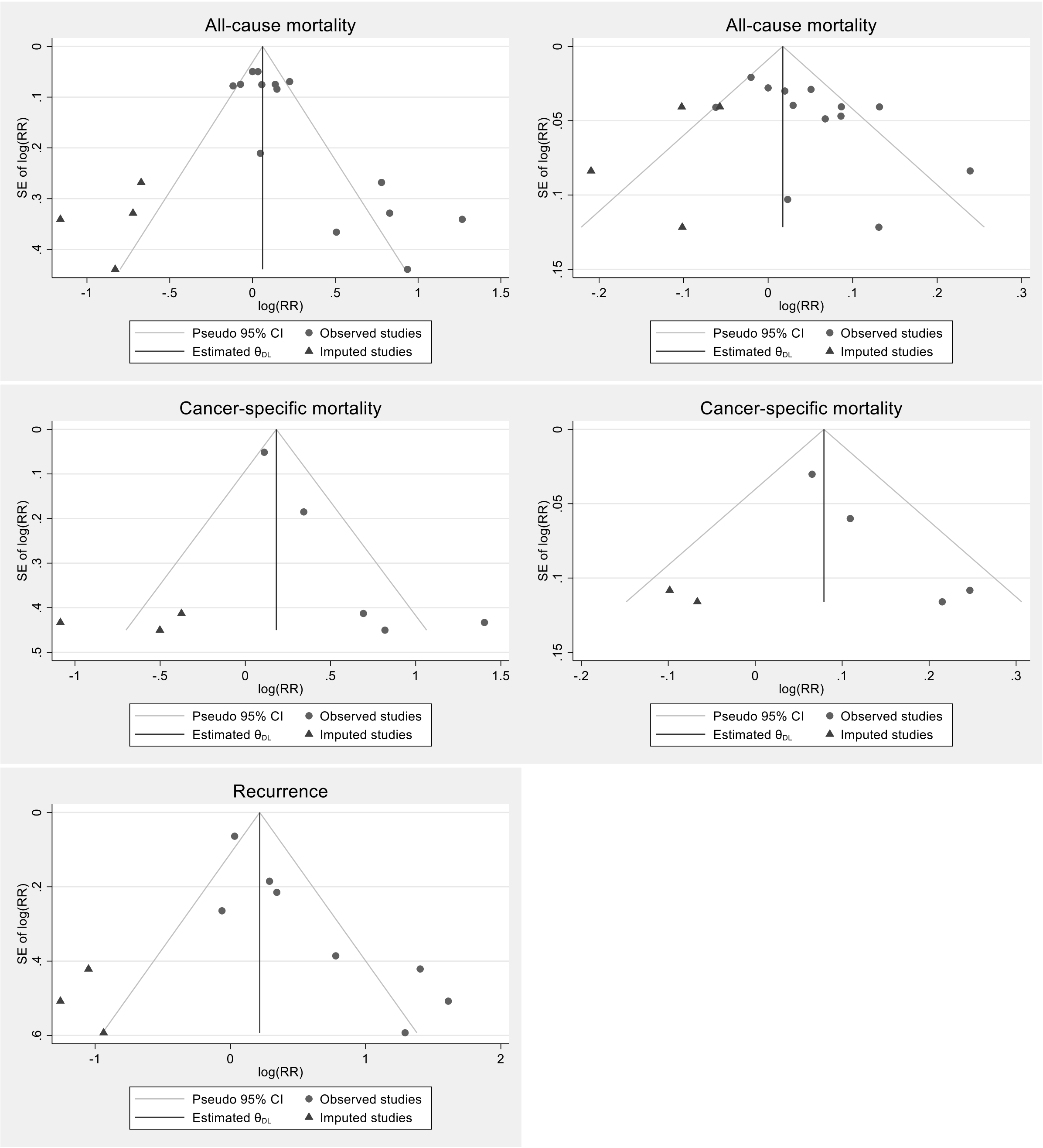

Left: Poorly controlled HbA_{1c} (≥7%) compared to well controlled HbA_{1c} (<7%);

Right: Per 1-unit increment of HbA_{1c} (%).

RR: Relative risk

Supplementary Figure 3. Meta-analysis within studies of high quality (NOS score ≥ 6)

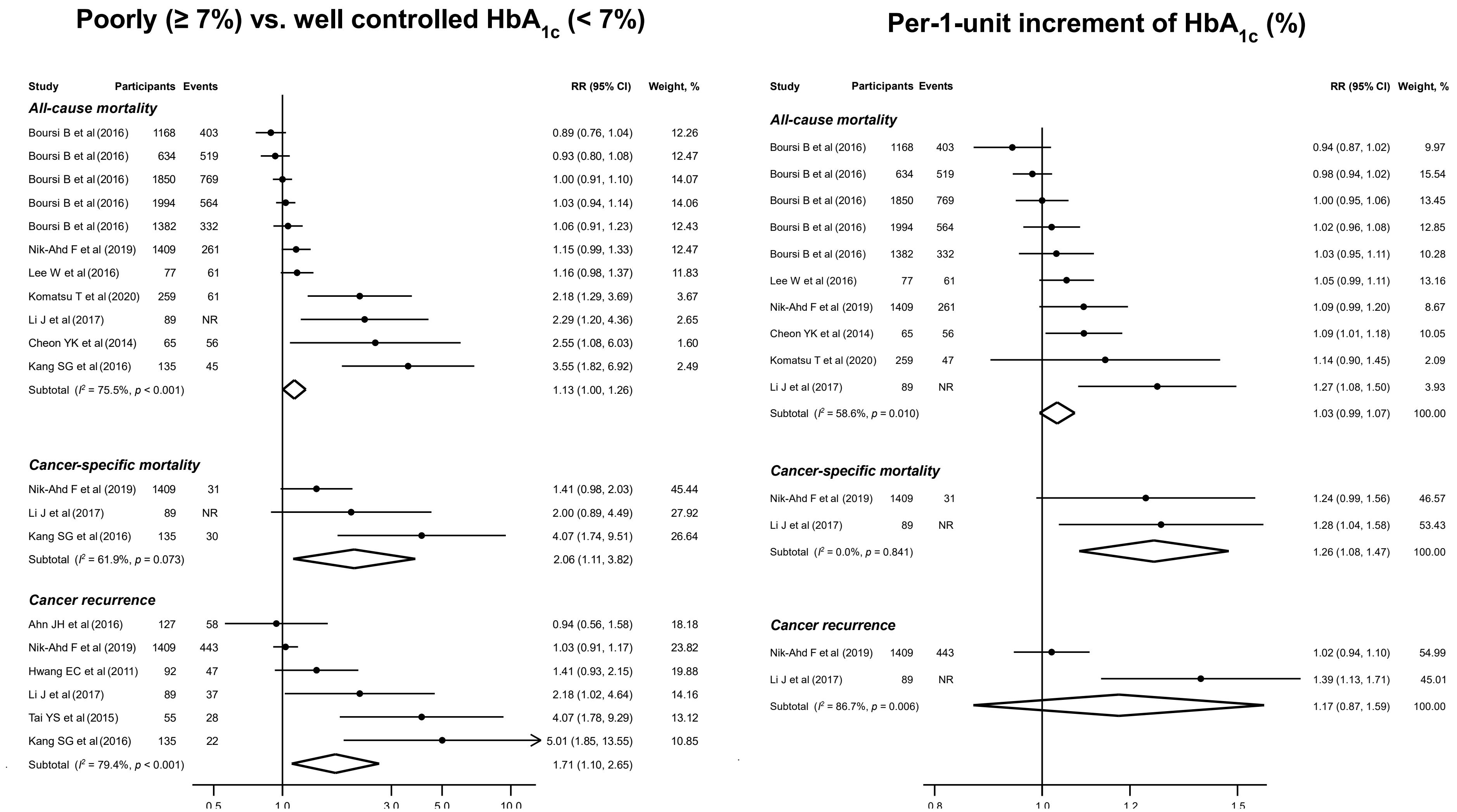

Supplementary Figure 4a. Subgroup analyses by geographical region for HbA_{1c} ≥ 7% vs. HbA_{1c} < 7%

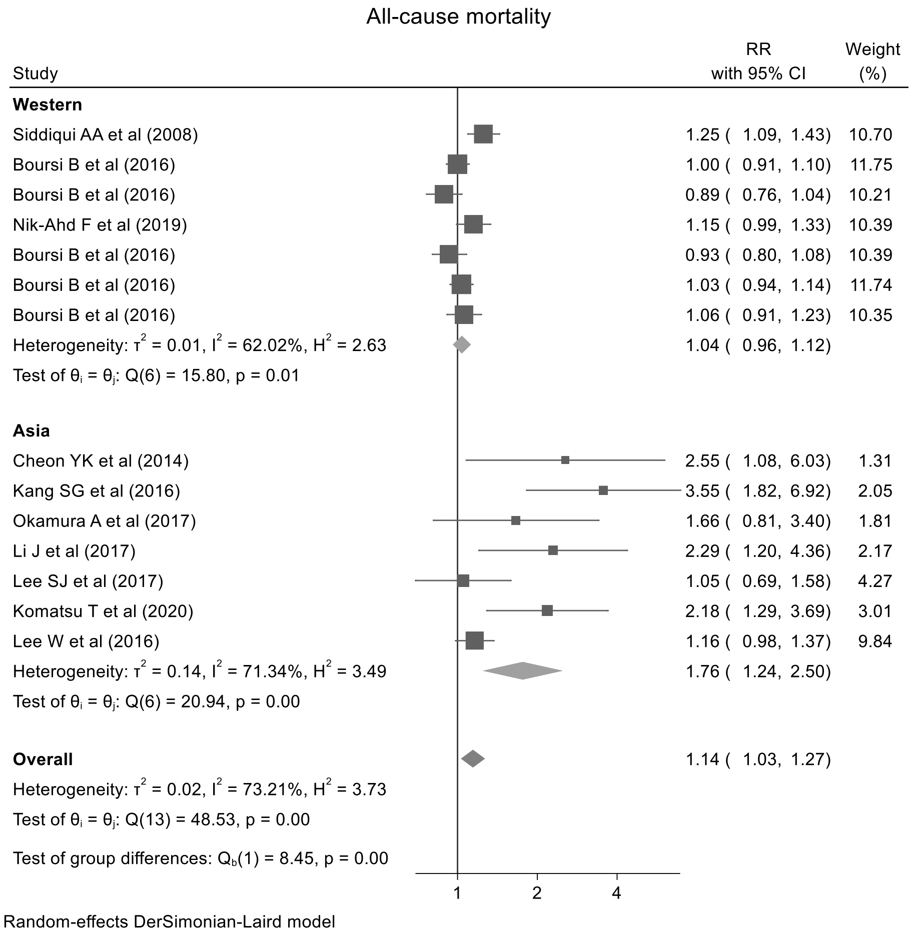

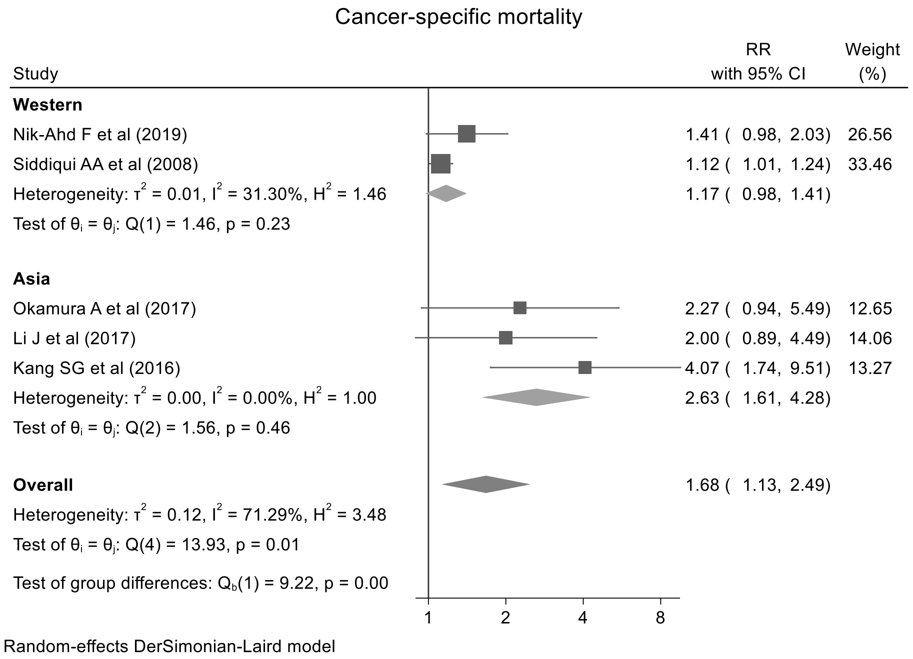

Supplementary Figure 4b. Subgroup analyses by geographical region per 1-unit increment of HbA_{1c}

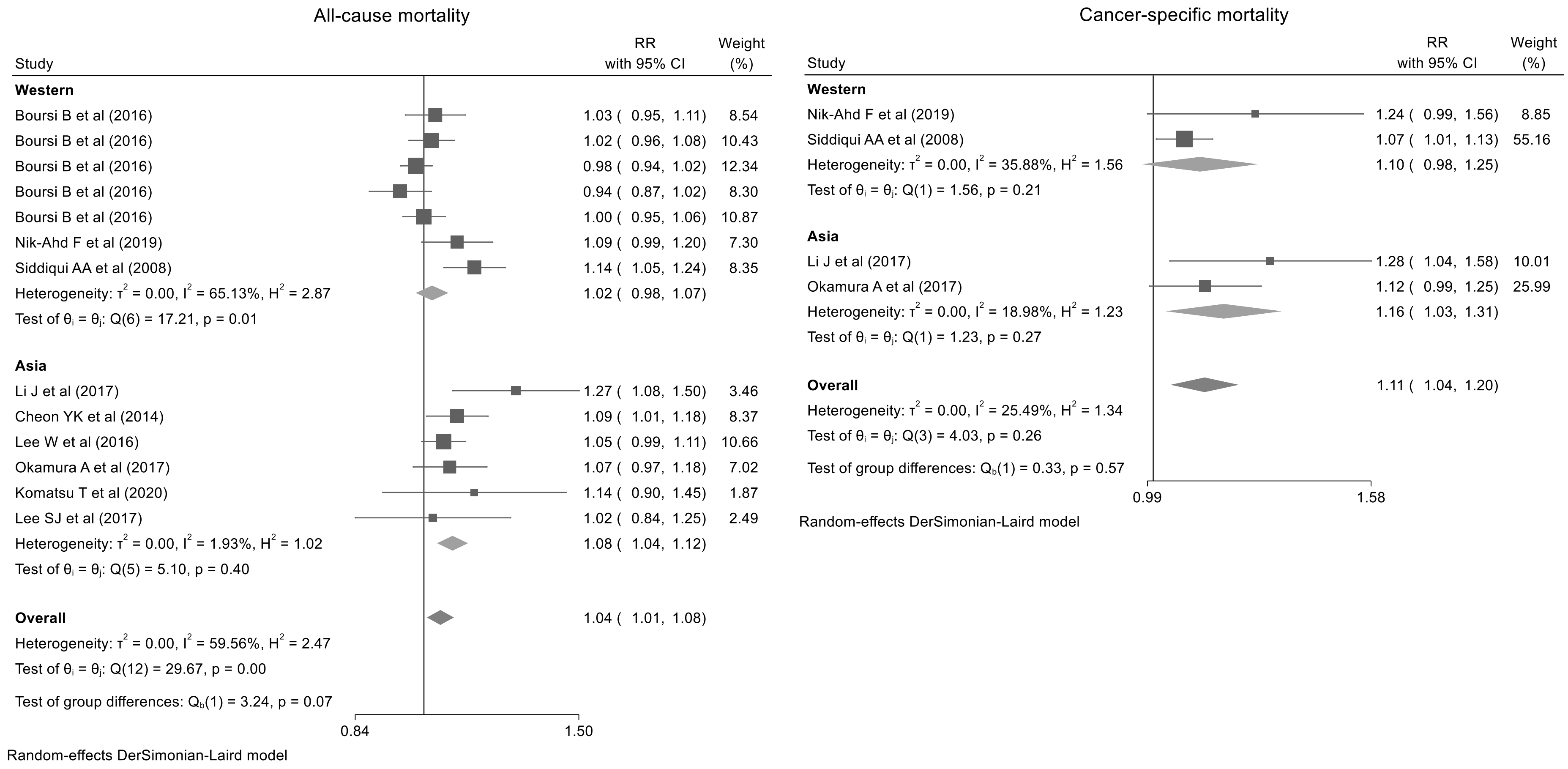

Supplementary Figure 5. Subgroup analyses by cancer sites

HbA_{1c} ≥ 7% vs. HbA_{1c} < 7%

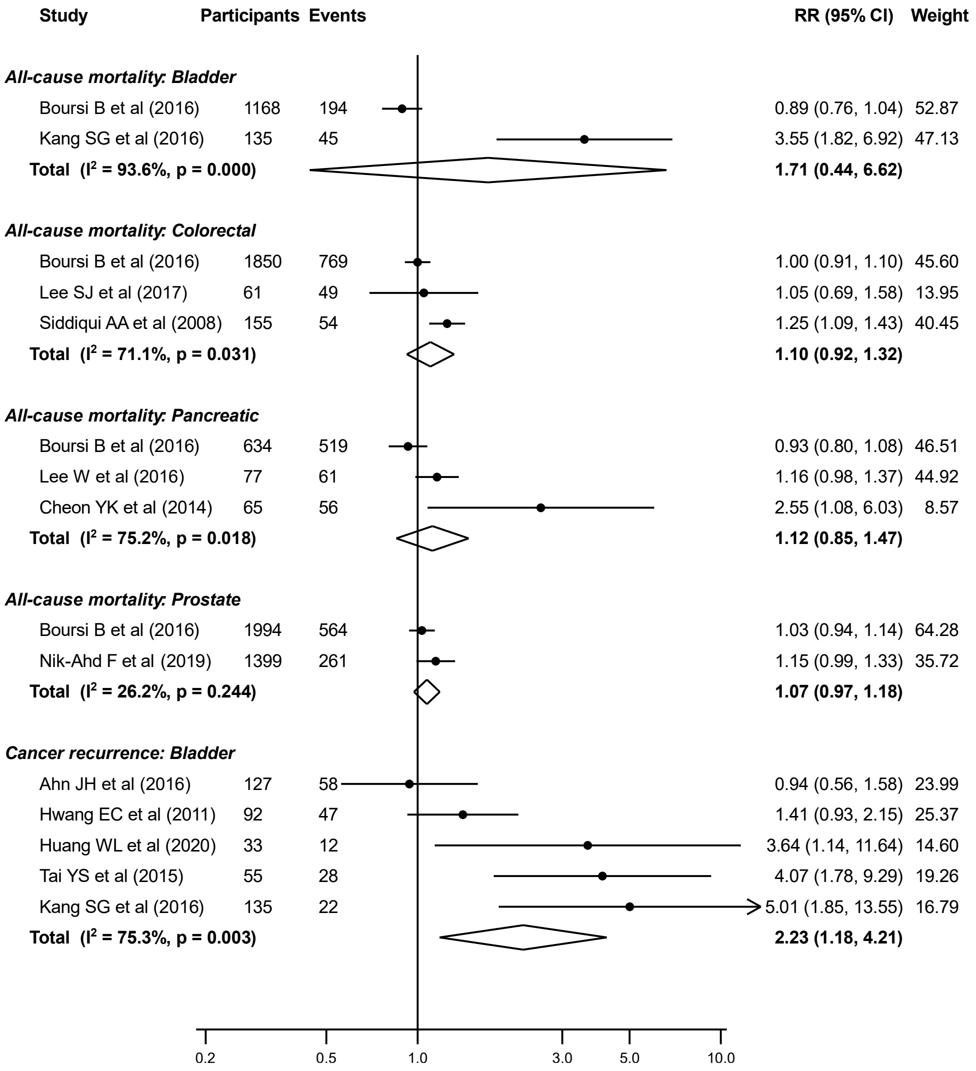

Per 1-unit increment of HbA_{1c} (%)

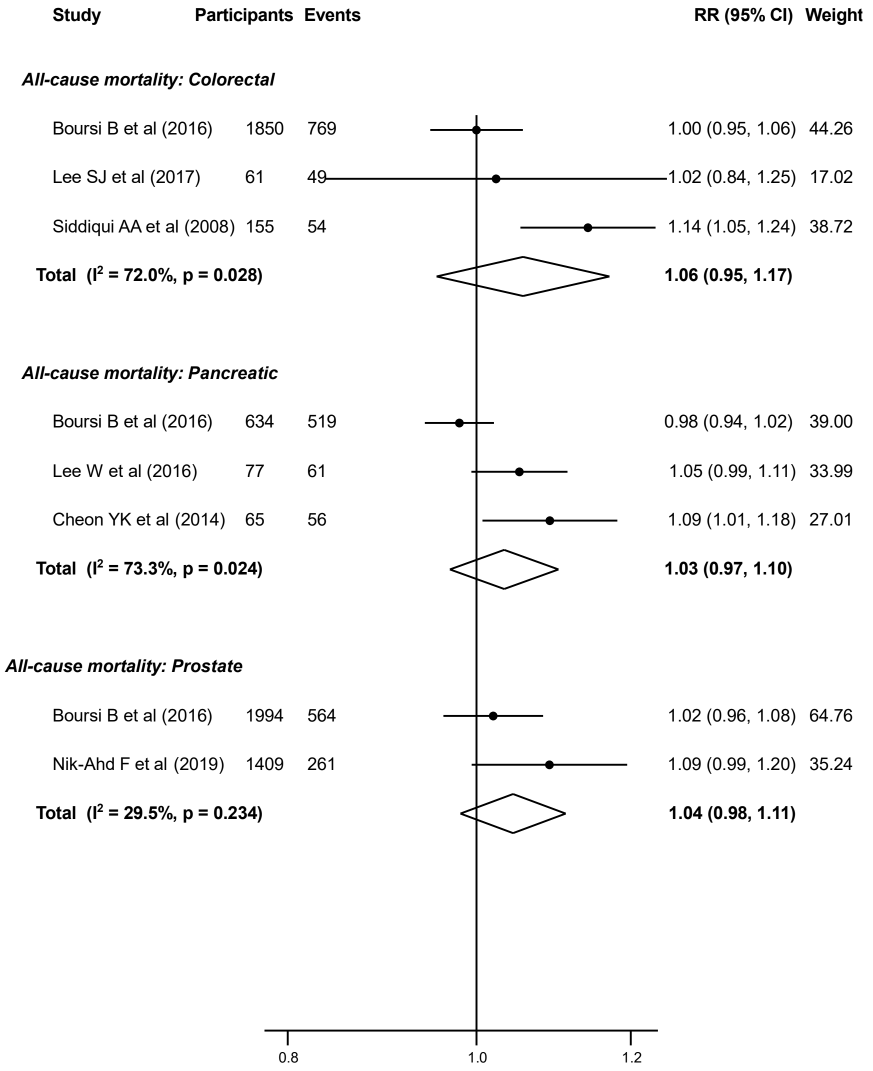

PRISMA checklist

Section and Topic	Item #	Checklist item	Location where item is reported
**TITLE**			
Title	1	Identify the report as a systematic review.	1
**ABSTRACT**			
Abstract	2	See the PRISMA 2020 for Abstracts checklist.	3
**INTRODUCTION**			
Rationale	3	Describe the rationale for the review in the context of existing knowledge.	4
Objectives	4	Provide an explicit statement of the objective(s) or question(s) the review addresses.	4
**METHODS**			
Eligibility criteria	5	Specify the inclusion and exclusion criteria for the review and how studies were grouped for the syntheses.	5
Information sources	6	Specify all databases, registers, websites, organisations, reference lists and other sources searched or consulted to identify studies. Specify the date when each source was last searched or consulted.	5
Search strategy	7	Present the full search strategies for all databases, registers and websites, including any filters and limits used.	5
Selection process	8	Specify the methods used to decide whether a study met the inclusion criteria of the review, including how many reviewers screened each record and each report retrieved, whether they worked independently, and if applicable, details of automation tools used in the process.	5
Data collection process	9	Specify the methods used to collect data from reports, including how many reviewers collected data from each report, whether they worked independently, any processes for obtaining or confirming data from study investigators, and if applicable, details of automation tools used in the process.	5
Data items	10a	List and define all outcomes for which data were sought. Specify whether all results that were compatible with each outcome domain in each study were sought (e.g. for all measures, time points, analyses), and if not, the methods used to decide which results to collect.	5
	10b	List and define all other variables for which data were sought (e.g. participant and intervention characteristics, funding sources). Describe any assumptions made about any missing or unclear information.	5
Study risk of bias assessment	11	Specify the methods used to assess risk of bias in the included studies, including details of the tool(s) used, how many reviewers assessed each study and whether they worked independently, and if applicable, details of automation tools used in the process.	6
Effect measures	12	Specify for each outcome the effect measure(s) (e.g. risk ratio, mean difference) used in the synthesis or presentation of results.	5-7
Synthesis methods	13a	Describe the processes used to decide which studies were eligible for each synthesis (e.g. tabulating the study intervention characteristics and comparing against the planned groups for each synthesis (item #5)).	5-7
	13b	Describe any methods required to prepare the data for presentation or synthesis, such as handling of missing summary statistics, or data conversions.	5-7
	13c	Describe any methods used to tabulate or visually display results of individual studies and syntheses.	5-7
	13d	Describe any methods used to synthesize results and provide a rationale for the choice(s). If meta-analysis was performed, describe the model(s), method(s) to identify the presence and extent of statistical heterogeneity, and software package(s) used.	5-7
	13e	Describe any methods used to explore possible causes of heterogeneity among study results (e.g. subgroup	6

Section and Topic	Item #	Checklist item	Location where item is reported
		analysis, meta-regression).	
	13f	Describe any sensitivity analyses conducted to assess robustness of the synthesized results.	6
Reporting bias assessment	14	Describe any methods used to assess risk of bias due to missing results in a synthesis (arising from reporting biases).	6
Certainty assessment	15	Describe any methods used to assess certainty (or confidence) in the body of evidence for an outcome.	5
**RESULTS**			
Study selection	16a	Describe the results of the search and selection process, from the number of records identified in the search to the number of studies included in the review, ideally using a flow diagram.	8
	16b	Cite studies that might appear to meet the inclusion criteria, but which were excluded, and explain why they were excluded.	Supplemental material
Study characteristics	17	Cite each included study and present its characteristics.	Supplemental material
Risk of bias in studies	18	Present assessments of risk of bias for each included study.	8, Supplemental material
Results of individual studies	19	For all outcomes, present, for each study: (a) summary statistics for each group (where appropriate) and (b) an effect estimate and its precision (e.g. confidence/credible interval), ideally using structured tables or plots.	8-10, Figure 2
Results of syntheses	20a	For each synthesis, briefly summarise the characteristics and risk of bias among contributing studies.	8-10, Figure 2
	20b	Present results of all statistical syntheses conducted. If meta-analysis was done, present for each the summary estimate and its precision (e.g. confidence/credible interval) and measures of statistical heterogeneity. If comparing groups, describe the direction of the effect.	8-10, Supplemental material
	20c	Present results of all investigations of possible causes of heterogeneity among study results.	9, Supplemental material
	20d	Present results of all sensitivity analyses conducted to assess the robustness of the synthesized results.	9, Supplemental material
Reporting biases	21	Present assessments of risk of bias due to missing results (arising from reporting biases) for each synthesis assessed.	8-10, Supplemental material
Certainty of evidence	22	Present assessments of certainty (or confidence) in the body of evidence for each outcome assessed.	8-10, Supplemental material
**DISCUSSION**			
Discussion	23a	Provide a general interpretation of the results in the context of other evidence.	11
	23b	Discuss any limitations of the evidence included in the review.	13-14
	23c	Discuss any limitations of the review processes used.	13-14
	23d	Discuss implications of the results for practice, policy, and future research.	11-14
**OTHER INFORMATION**			
Registration and protocol	24a	Provide registration information for the review, including register name and registration number, or state that the review was not registered.	5
	24b	Indicate where the review protocol can be accessed, or state that a protocol was not prepared.	5

Section and Topic	Item #	Checklist item	Location where item is reported
	24c	Describe and explain any amendments to information provided at registration or in the protocol.	NA
Support	25	Describe sources of financial or non-financial support for the review, and the role of the funders or sponsors in the review.	15
Competing interests	26	Declare any competing interests of review authors.	15
Availability of data, code and other materials	27	Report which of the following are publicly available and where they can be found: template data collection forms; data extracted from included studies; data used for all analyses; analytic code; any other materials used in the review.	15

References

[1] Alpertunga I, Sadiq R, Pandya D, Lo T, Dulgher M, Evans S, et al. Glycemic Control as an Early Prognostic Marker in Advanced Pancreatic Cancer. Frontiers in Oncology. 2021;11.

[2] Arrieta O, Varela-Santoyo E, Soto-Perez-de-Celis E, Sanchez-Reyes R, De la Torre-Vallejo M, Muniz-Hernandez S, et al. Metformin use and its effect on survival in diabetic patients with advanced non-small cell lung cancer. Bmc Cancer. 2016;16.

[3] Barua R, Templeton AJ, Seruga B, Ocana A, Amir E, Ethier JL. Hyperglycaemia and Survival in Solid Tumours: A Systematic Review and Meta-analysis. Clin Oncol (R Coll Radiol). 2018;30:215-24.

[4] Cantiello F, Cicione A, Salonia A, Autorino R, De Nunzio C, Briganti A, et al. Association between metabolic syndrome, obesity, diabetes mellitus and oncological outcomes of bladder cancer: a systematic review. Int J Urol. 2015;22:22-32.

[5] Chang YL, Sheu WHH, Lin SY, Liou WS. Good glycaemic control is associated with a better prognosis in breast cancer patients with type 2 diabetes mellitus. Clinical and Experimental Medicine. 2018;18:383-90.

[6] Chen S, Tao M, Zhao L, Zhang X. The association between diabetes/hyperglycemia and the prognosis of cervical cancer patients: A systematic review and meta-analysis. Medicine (Baltimore). 2017;96:e7981.

[7] Chen XH, Chen YH, Li T, Jun L, Lin T, Hu YF, et al. Impact of diabetes on prognosis of gastric cancer patients performed with gastrectomy. Chinese Journal of Cancer Research. 2020;32:631-+.

[8] Chia CLK, Lee AYS, Shelat VG, Ahmed S, Junnarkar SP, Woon WWL, et al. Does diabetes mellitus affect presentation, stage and survival in operable pancreatic cancer? Hepatobiliary Surgery and Nutrition. 2016;5:38-42.

[9] Choe S, Lee J, Park JW, Jeong SY, Cho YM, Park BJ, et al. Prognosis of Patients with Colorectal Cancer with Diabetes According to Medication Adherence: A Population-Based Cohort Study. Cancer Epidemiology Biomarkers & Prevention. 2020;29:1120-7.

[10] Connor AE, Visvanathan K, Boone SD, Rifai N, Baumgartner KB, Baumgartner RN. Fructosamine and diabetes as predictors of mortality among Hispanic and non-Hispanic white breast cancer survivors. Npj Breast Cancer. 2019;5.

[11] Ederaine SA, Dominguez JL, Harvey JA, Mangold AR, Cook CB, Kosiorek H, et al. Survival and glycemic control in patients with co-existing squamous cell carcinoma and diabetes mellitus. Future Science Oa. 2021;7.

[12] Erickson K, Patterson RE, Flatt SW, Natarajan L, Parker BA, Heath DD, et al. Clinically defined type 2 diabetes mellitus and prognosis in early-stage breast cancer. J Clin Oncol. 2011;29:54-60.

[13] Fan KY, Dholakia AS, Wild AT, Su Z, Hacker-Prietz A, Kumar R, et al. Baseline Hemoglobin-A1c Impacts Clinical Outcomes in Patients With Pancreatic Cancer. Journal of the National Comprehensive Cancer Network. 2014;12:50-7.

[14] Ferroni P, Formica V, Della-Morte D, Lucchetti J, Spila A, D'Alessandro R, et al. Prognostic value of glycated hemoglobin in colorectal cancer. World Journal of Gastroenterology. 2016;22:9984-93.

[15] Hope C, Robertshaw A, Cheung KL, Idris I, English E. Relationship between HbA1c and cancer in people with or without diabetes: a systematic review. Diabet Med. 2016;33:1013-25.

[16] Hershey DS, Hession S. Chemotherapy and Glycemic Control in Patients with Type 2 Diabetes and Cancer: A Comparative Case Analysis. Asia Pac J Oncol Nurs. 2017;4:224-32.

[17] Iavazzo C, McComiskey M, Datta M, Ryan M, Kiernan J, Winter-Roach B, et al. Preoperative HBA1c and risk of postoperative complications in patients with gynaecological cancer. Arch Gynecol Obstet. 2016;294:161-4.

[18] Joshu CE, Prizment AE, Dluzniewski PJ, Menke A, Folsom AR, Coresh J, et al. Glycated hemoglobin and cancer incidence and mortality in the Atherosclerosis in Communities (ARIC) Study, 1990-2006. Int J Cancer. 2012;131:1667-77.

[19] Karlin NJ, Amin SB, Buras MR, Kosiorek HE, Verona PM, Cook CB. Patient outcomes from lung cancer and diabetes mellitus: a matched case-control study. Future Science Oa. 2018;4.

[20] Karlin NJ, Amin SB, Kosiorek HE, Buras MR, Verona PM, Cook CB. Survival and glycemic control in patients with colorectal cancer and diabetes mellitus. Future Science Oa. 2018;4.

[21] Karlin NJ, Amin SB, Kosiorek HE, Buras MR, Verona PM, Cook CB. Survival and glycemic control outcomes among patients with coexisting pancreatic cancer and diabetes mellitus. Future Science Oa. 2018;4.

[22] Karlin NJ, Amin SB, Verona PM, Kosiorek HE, Cook CB. CO-EXISTING PROSTATE CANCER AND DIABETES MELLITUS: IMPLICATIONS FOR PATIENT OUTCOMES AND CARE. Endocrine Practice. 2017;23:816-21.

[23] Karlin NJ, Buras MR, Kosiorek HE, Coppola KE, Verona PM, Cook CB. Assessing the relationship between institutional cancer and diabetes mortality rates using National Death Index data. Future Science Oa. 2020;6.

[24] Karlin NJ, Buras MR, Kosiorek HE, Verona PM, Cook CB. Glycemic control and survival of patients with coexisting diabetes mellitus and gastric or esophageal cancer. Future Science Oa. 2019;5.

[25] Karlin NJ, Dueck AC, Cook CB. Cancer with diabetes: prevalence, metabolic control, and survival in an academic oncology practice. Endocr Pract. 2012;18:898-905.

[26] Karlin NJ, Mangold AR, Amin SB, Kosiorek HE, Buras MR, Verona PM, et al. Survival and glycemic control in patients with coexisting melanoma and diabetes mellitus. Future Science Oa. 2019;5.

[27] Kaseda K, Hishida T, Masai K, Asakura K, Hayashi Y, Asamura H. Clinicopathological and prognostic features of operable non-small cell lung cancer patients with diabetes mellitus. Journal of Surgical Oncology. 2020.

[28] Kim HS, Presti JC, Jr., Aronson WJ, Terris MK, Kane CJ, Amling CL, et al. Glycemic control and prostate cancer progression: results from the SEARCH database. Prostate. 2010;70:1540-6.

[29] Kochi R, Suzuki T, Yajima S, Oshima Y, Ito M, Funahashi K, et al. Does Preoperative Low HbA1c Predict Esophageal Cancer Outcomes? Annals of Thoracic and Cardiovascular Surgery. 2020;26:184-9.

[30] Kondo S, Kondo M, Kondo A. Glycemia control using A1C level in terminal cancer patients with preexisting type 2 diabetes. J Palliat Med. 2013;16:790-3.

[31] Kurishima K, Watanabe H, Ishikawa H, Satoh H, Hizawa N. Survival of patients with lung cancer and diabetes mellitus. Molecular and Clinical Oncology. 2017;6:907-10.

[32] Lee H, Kuk H, Byun SS, Lee SE, Hong SK. Preoperative Glycemic Control Status as a Significant Predictor of Biochemical Recurrence in Prostate Cancer Patients after Radical Prostatectomy. Plos One. 2015;10.

[33] Lee H, Kwak C, Kim HH, Byun SS, Lee SE, Hong SK. Diabetes Mellitus as an Independent Predictor of Survival of Patients Surgically Treated for Renal Cell Carcinoma: A Propensity Score Matching Study. Journal of Urology. 2015;194:1554-60.

[34] Li J, Liu Y, Zhang H, Hua H. Association between hyperglycemia and the malignant transformation of oral leukoplakia in China. Oral Dis. 2020;26:1402-13.

[35] Liang SH, Shen YC, Wu JY, Wang LJ, Wu MF, Li J. Impact of Poor Preoperative Glycemic Control on Outcomes among Patients with Cervical Cancer Undergoing a Radical Hysterectomy. Oncology Research and Treatment. 2020;43:10-8.

[36] Liu H, Liu Z, Jiang B, Ding X, Huo L, Wan X, et al. Prognostic Significance of Hyperglycemia in Patients with Brain Tumors: a Meta-Analysis. Mol Neurobiol. 2016;53:1654-60.

[37] Motoishi M, Sawai S, Hori T, Yamashita N. The preoperative HbA1c level is an independent prognostic factor for the postoperative survival after resection of non-small cell lung cancer in elderly patients. Surgery Today. 2018;48:517-24.

[38] Murtola TJ, Salli SM, Talala K, Taari K, TammelaL TLJ, Auvinen A. Blood glucose, glucose balance, and disease-specific survival after prostate cancer diagnosis in the Finnish Randomized Study of Screening for Prostate Cancer. Prostate Cancer and Prostatic Diseases. 2019;22:453-60.

[39] Onitilo AA, Donald M, Stankowski RV, Engel JM, Williams G, Doi SA. Breast and prostate cancer survivors in a diabetic cohort: results from the Living with Diabetes Study. Clin Med Res. 2013;11:210-8.

[40] Ogawa H, Fujibayashi Y, Nishikubo M, Nishioka Y, Tane S, Kitamura Y, et al. Prognostic significance of preoperative haemoglobin A1c level in patients with lung adenocarcinoma. Interact Cardiovasc Thorac Surg. 2021;33:534-40.

[41] Pusceddu S, Vernieri C, Di Maio M, Marconcini R, Spada F, Massironi S, et al. Metformin Use Is Associated With Longer Progression-Free Survival of Patients With Diabetes and Pancreatic Neuroendocrine Tumors Receiving Everolimus and/or Somatostatin Analogues. Gastroenterology. 2018;155:479-+.

[42] Sandini M, Strobel O, Hank T, Lewosinska M, Niessen A, Hackert T, et al. Pre-operative dysglycemia is associated with decreased survival in patients with pancreatic neuroendocrine neoplasms. Surgery. 2020;167:575-80.

[43] Shi HJ, Jin C, Fu DL. Impact of postoperative glycemic control and nutritional status on clinical outcomes after total pancreatectomy. World Journal of Gastroenterology. 2017;23:265-74.

[44] Shimada S, Kamiyama T, Orimo T, Nagatsu A, Kamachi H, Taketomi A. High HbA1c is a risk factor for complications after hepatectomy and influences for hepatocellular carcinoma without HBV and HCV infection. Hepatobiliary Surgery and Nutrition. 2021;10:454-63.

[45] Simon JM, Thomas F, Czernichow S, Hanon O, Lemogne C, Simon T, et al. Hyperglycaemia is associated with cancer-related but not non-cancer-related deaths: evidence from the IPC cohort. Diabetologia. 2018;61:1089-97.

[46] Suceveanu AI, Suceveanu AP, Parepa I, Ardeleanu V, Micu IS, Dumitru A, et al. Uncontrolled Diabetes Mellitus - Negative Predictive Factor for Colorectal Cancer Recurrence after Curative Surgical Treatment. 6th International Conference on Interdisciplinary Management of Diabetes Mellitus and its Complications (INTERDIAB). Bucharest, ROMANIA2020. p. 74-80.

[47] van Herpt TTW, van de Schans SAM, Haak HR, van Spronsen DJ, Dercksen MW, Janssen-Heijnen MLG. Treatment and outcome in non-Hodgkin's lymphoma patients with and without prevalent diabetes mellitus in a population-based cancer registry. Journal of Geriatric Oncology. 2011;2:239-45.

[48] Wrenn SM, Pandian TK, Gartland RM, Fong ZV, Nehs MA. Diabetes mellitus and hyperglycemia are associated with inferior oncologic outcomes in adrenocortical carcinoma. Langenbecks Arch Surg. 2021;406:1599-606.

[49] Zeng XH, Xu C, Cheng JN, Sun CD, Wang ZY, Gong ZH, et al. Poor glycemic control might compromise the efficacy of chemotherapy in non-small cell lung cancer patients with diabetes mellitus. Cancer Medicine. 2020;9:902-11.

[50] Zhao XB, Ren GS. Diabetes mellitus and prognosis in women with breast cancer: A systematic review and meta-analysis. Medicine (Baltimore). 2016;95:e5602.

[51] Ahn JH, Jung SI, Yim SU, Kim SW, Hwang EC, Kwon DD. Impact of Glycemic Control and Metformin Use on the Recurrence and Progression of Non-Muscle Invasive Bladder Cancer in Patients with Diabetes Mellitus. J Korean Med Sci. 2016;31:1464-71.

[52] Boursi B, Giantonio BJ, Lewis JD, Haynes K, Mamtani R, Yang YX. Serum glucose and hemoglobin A1C levels at cancer diagnosis and disease outcome. Eur J Cancer. 2016;59:90-8.

[53] Cheon YK, Koo JK, Lee YS, Lee TY, Shim CS. Elevated hemoglobin A1c levels are associated with worse survival in advanced pancreatic cancer patients with diabetes. Gut Liver. 2014;8:205-14.

[54] Huang WL, Huang KH, Huang CY, Pu YS, Chang HC, Chow PM. Effect of diabetes mellitus and glycemic control on the prognosis of non-muscle invasive bladder cancer: a retrospective study. BMC Urol. 2020;20:117.

[55] Hwang EC, Kim YJ, Hwang IS, Hwang JE, Jung SI, Kwon DD, et al. Impact of diabetes mellitus on recurrence and progression in patients with non-muscle invasive bladder carcinoma: a retrospective cohort study. Int J Urol. 2011;18:769-76.

[56] Kaneda K, Uenishi T, Takemura S, Shinkawa H, Urata Y, Sakae M, et al. The influence of postoperative glycemic control on recurrence after curative resection in diabetics with hepatitis C virus-related hepatocellular carcinoma. J Surg Oncol. 2012;105:606-11.

[57] Kang SG, Hwang EC, Jung SI, Yu HS, Chung HS, Kang TW, et al. Poor Preoperative Glycemic Control Is Associated with Dismal Prognosis after Radical Nephroureterectomy for Upper Tract Urothelial Carcinoma: A Korean Multicenter Study. Cancer Res Treat. 2016;48:1293-301.

[58] Komatsu T, Chen-Yoshikawa TF, Ikeda M, Takahashi K, Nishimura A, Harashima SI, et al. Impact of diabetes mellitus on postoperative outcomes in individuals with non-small-cell lung cancer: A retrospective cohort study. PLoS One. 2020;15:e0241930.

[59] Lee W, Yoon YS, Han HS, Cho JY, Choi Y, Jang JY, et al. Prognostic relevance of preoperative diabetes mellitus and the degree of hyperglycemia on the outcomes of resected pancreatic ductal adenocarcinoma. J Surg Oncol. 2016;113:203-8.

[60] Lee SJ, Kim JH, Park SJ, Ock SY, Kwon SK, Choi YS, et al. Optimal glycemic target level for colon cancer patients with diabetes. Diabetes Res Clin Pract. 2017;124:66-71.

[61] Li J, Ning NY, Rao QX, Chen R, Wang LJ, Lin ZQ. Pretreatment glycemic control status is an independent prognostic factor for cervical cancer patients receiving neoadjuvant chemotherapy for locally advanced disease. BMC Cancer. 2017;17:517.

[62] Nik-Ahd F, Howard LE, Eisenberg AT, Aronson WJ, Terris MK, Cooperberg MR, et al. Poorly controlled diabetes increases the risk of metastases and castration-resistant prostate cancer in men undergoing radical prostatectomy: Results from the SEARCH database. Cancer. 2019;125:2861-7.

[63] Okamura A, Watanabe M, Imamura Y, Hayami M, Yamashita K, Kurogochi T, et al. Glycemic Status and Prognosis of Patients with Squamous Cell Carcinoma of the Esophagus. World J Surg. 2017;41:2591-7.

[64] Siddiqui AA, Spechler SJ, Huerta S, Dredar S, Little BB, Cryer B. Elevated HbA1c is an independent predictor of aggressive clinical behavior in patients with colorectal cancer: a case-control study. Dig Dis Sci. 2008;53:2486-94.

[65] Tai YS, Chen CH, Huang CY, Tai HC, Wang SM, Pu YS. Diabetes mellitus with poor glycemic control increases bladder cancer recurrence risk in patients with upper urinary tract urothelial carcinoma. Diabetes Metab Res Rev. 2015;31:307-14.
